# Supplementary material for: Anti-SARS-CoV-2 serology based on ancestral RBD antigens does not correlate with the presence of neutralizing antibodies against Omicron variants
Source: Microbiol Spectr. 2024 Nov 20;13(1):e01568-24. doi: 10.1128/spectrum.01568-24 (PMC11705886; doi:10.1128/spectrum.01568-24)
Supplement: Figure S1 — Neutralizing antibodies titers against the Wuhan, BA.2, BA.4/5, BQ1.1, and XBB.1 viruses in patients infected in 2020 and during the successive Omicron waves. [file spectrum.01568-24-s0001.docx]

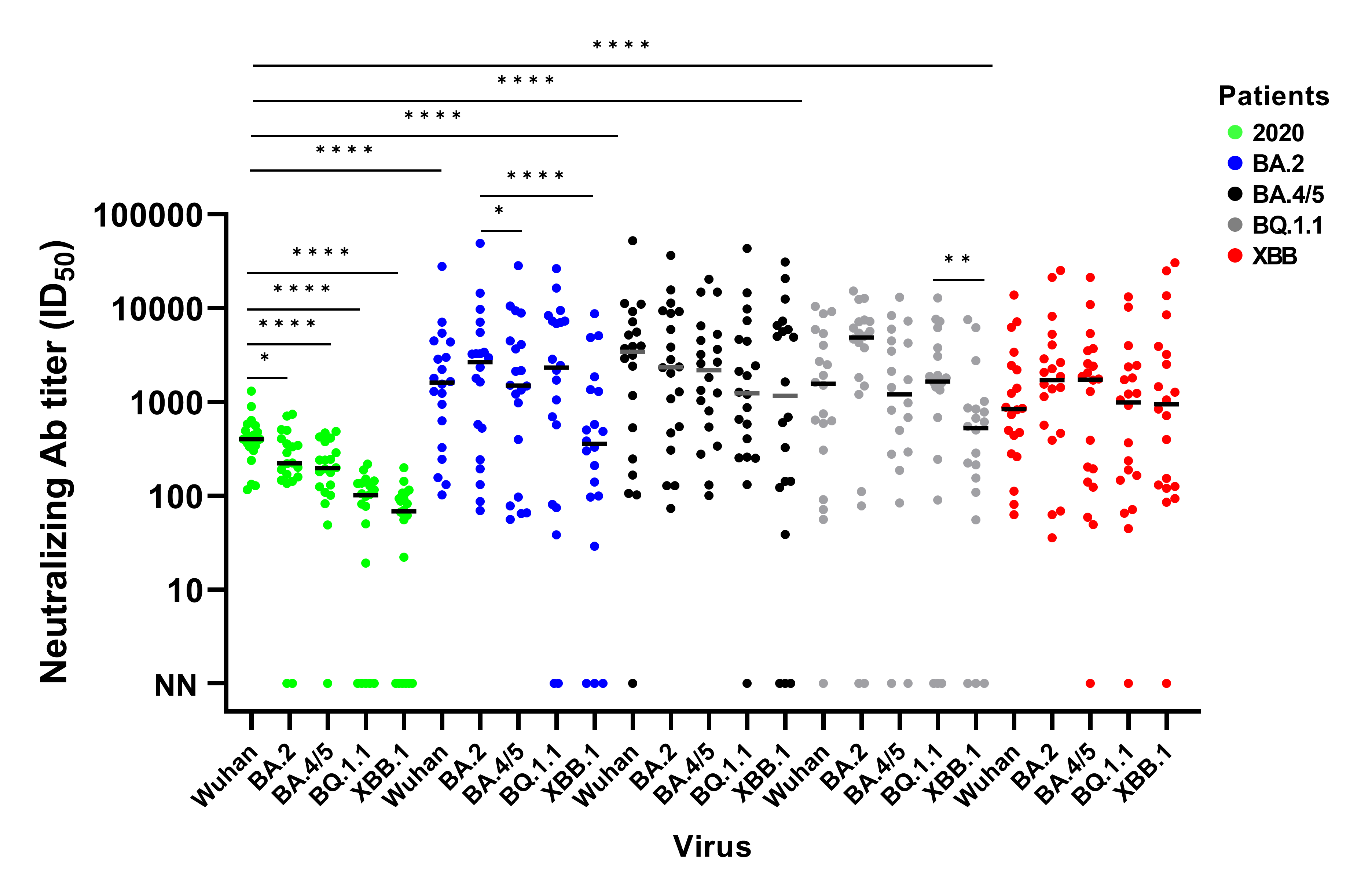


**Figure S1**: Neutralizing antibodies titers against the Wuhan, BA.2, BA.4/5, BQ1.1, and XBB.1 viruses in patients infected in 2020 and during the successive Omicron waves (median titer (median titers for BA.5 patients are shown in grey for easier viewing). Antibody titers were compared using the non-parametric Wilcoxon signed-rank test (paired samples) or Wilcoxon rank-sum test when appropriate (independent samples). * p< 0.05; ** p<0.01; **** p< 0.0001.
