# Supplementary material for: Anti-SARS-CoV-2 serology based on ancestral RBD antigens does not correlate with the presence of neutralizing antibodies against Omicron variants
Source: Microbiol Spectr. 2024 Nov 20;13(1):e01568-24. doi: 10.1128/spectrum.01568-24 (PMC11705886; doi:10.1128/spectrum.01568-24)
Supplement: Supplemental tables — Tables S1 to S3. [file spectrum.01568-24-s0002.docx]

**Table S1**: Characteristics of the patients

|  | Wuhan  n=20 | BA.2  n=20 | BA.4/5  n=20 | BQ.1.1  n=20 | XBB  n=20 |
| --- | --- | --- | --- | --- | --- |
| Sex, male (%) | 70 | 45 | 60 | 70 | 45 |
| Median age [IQR] | 67  [58.25-70.75] | 77  [62-86.5] | 67  [53.25-82.75] | 74  [63.5-83.25] | 74.5  [49.25-85] |
| Median delay between collection and PCR diagnosis, day [IQR] | 170  [165-190] | 36  [26-42] | 38.5  [25.25-51.25] | 34  [26-40] | 24  [22-27] |
| Strain sequencing yes/No (No.) | No | yes | yes | yes | Yes  XBB (3)  XBB.1.5 (9)  XBB.1.9 (8) |
| Vaccination with two or more doses  (BNT 162b2 or mRNA1273 or chAdOx1 + BNT 162b2/mRNA1273)  Adapted bivalent vaccines (including Omicron BA4/5 valence) were administered from autumn 2022 (for the fourth or more dose) (No.)  Vaccination not documented (No.) | 0  NA | 16  4 | 17  3 | 16  4 | 12  6 |
| Origin of the patients |  |  |  |  |  |
| Non-hospitalized patients (n=62):  Dialysis patients (n=35)  Transplant patients (n=17)  Gynecology consultation (n=3)  Infectious disease consultation (n=2)  Health care professionals (n=5) | 0 | 16  9  5  1  0  1 | 14  8  5  1  0  0 | 17  11  3  0  1  2 | 15  7  4  1  1  2 |
| Hospitalized patients (n=38) | 20 | 4 | 6 | 3 | 5 |

IQR: interquartile range; N/A: not applicable; PCR: polymerase chain reaction

**Table S2**: Spearman’s correlation between serology and neutralization assays against Wuhan, BA.2, BA4/5, BQ.1.1, and XBB.1 viruses

| Spearman’s correlation  [CI 95%] | Spike ELISA | RBD ELISA | Spike-ΔRBD ELISA | VIDAS^®^ assay |
| --- | --- | --- | --- | --- |
| PV Wuhan | 0.71  [0.60 - 0.80] | 0.73  [0,62 - 0,81] | 0.50  [0,33 - 0,64] | 0.76  [0,66 - 0,83] |
| PV BA.2 | 0.77  [0.67 - 0.84] | 0.77  [0,67 - 0,84] | 0.57  [0,41 - 0,69] | 0.82  [0,74 - 0,88] |
| PV BA.4/5 | 0.75  [0.64 - 0.83] | 0.73  [0,62 - 0,81] | 0.68  [0,55 - 0,78] | 0.77  [0,67 - 0,84] |
| PV BQ.1.1 | 0.60  [0.45 - 0.71] | 0.59  [0,43 - 0,70] | 0.61  [0,47 - 0,72] | 0.63  [0,49 - 0,74] |
| PV XBB.1 | 0.64  [0.50 - 0.74] | 0.63  [0,49 - 0,74] | 0.54  [0,38 - 0,67] | 0.61  [0,46 - 0,72] |

Rs: Spearman’s correlation; CI 95%: confidence interval; PV: Pseudovirus

**Table S3**: Performance values of VIDAS^®^ assay and Spike ΔRBD ELISA for detecting neutralizing antibodies against the Wuhan, B.2, BA4/5, BQ1.1, and XBB.1 viruses

|  | | Pseudo-typed virus | | | | |
| --- | --- | --- | --- | --- | --- | --- |
|  | | Wuhan | BA.2 | BA.4/5 | BQ1.1 | XBB.1 |
| VIDAS^®^ assay  Provider’s cut-off set at 25 BAU/mL | Sens  95% CI | 0.97  0.91-0.99 | 0.97  0.91-0.99 | 0.97  0.91-0.99 | **0.97**  **0.90-0.99** | **0.98**  **0.91-1.00** |
|  | Spe  95% CI | 1  0.16-1.00 | 0.50  0.07-0.93 | 0.50  0.07-0.93 | **0.15**  **0.02-0.45** | **0.17**  **0.04-0.41** |
|  | NPV  95% CI | 0.40  0.05-0.85 | 0.40  0.05-0.85 | 0.40  0.05-0.85 | 0.40  0.05-0.85 | 0.60  0.15-0.95 |
|  | PPV  95% CI | 1  0.96-1.00 | 0.98  0.93-1.00 | 0.98  0.93-1.00 | **0.88**  **0.80-0.94** | **0.84**  **0.75-0.91** |
|  | Agreement: κ  95% CI  Interpretation* | 0.56  0.11-1.00  strong | 0.42  0-0.84  moderate | 0.42  0-0.84  moderate | 0.16  0-0.43  very low | 0.20  0-0.43  very low |
| VIDAS^®^ assay  Optimized cut-off according to the virus tested | AUC | 0.99 | 0.96 | 0.91 | 0.77 | 0.81 |
|  | Cut Off | 13.5 | 157 | 96.25 | 4324.5 | 865.5 |
|  | Sens  95% CI | 0.99  0.94-1.00 | 0.90  0.82-0.95 | 0.94  0.87-0.98 | **0.53**  **0.42-0.64** | **0.71**  **0.60-0.80** |
|  | Spe  95% CI | 1  0.16-1.00 | 1  0.40-1.00 | 0.75  0.19-0.99 | **0.92**  **0.64-1.00** | **0.83**  **0.59-0.96** |
|  | NPV  95% CI | 0.67  0.9-0.99 | 0.29  0.08-0.58 | 0.33  0.07-0.70 | 0.23  0.12-0.36 | 0.38  0.23-0.55 |
|  | PPV  95% CI | 1  0.96-1.00 | 1  0.96-1.00 | 0.99  0.94-1.00 | **0.98**  **0.89-1.00** | **0.95**  **0.86-0.99** |
|  | Agreement: κ  95% CI  Interpretation* | 0.80  0.40-1.00  strong | 0.41  0.12-0.69  moderate | 0.43  0.08-0.78  moderate | 0.20  0.07-0.32  very low | 0.37  0.20-0.55  low |
| ΔRBD-S ELISA  Optimized cut-off according to the virus tested | AUC | 0.93 | 0.94 | 0.89 | 0.88 | 0.82 |
|  | Cut off | 2.85 | 2.85 | 33.09 | 26.92 | 84.56 |
|  | Sens  95% CI | 0.87  0.78-0.93 | 0.89  0.80-0.94 | 0.74  0.64-0.82 | 0.83  0.73-0.90 | 0.73  0.62-0.82 |
|  | Spe  95% CI | 1  0.16-1.00 | 1  0.40-1.00 | 1  0.40-1.00 | 0.85  0.55-0.98 | 0.89  0.65-0.99 |
|  | VPN  95% CI | 0.13  0.02-0.40 | 0.27  0.08-0.55 | 0.14  0.04-0.32 | 0.42  0.23-0.63 | 0.42  0.26-0.59 |
|  | VPP  95% CI | 1  0.96-1.00 | 1  0.96-1.00 | 1  0.95-1.00 | 0.97  0.91-1.00 | 0.97  0.89-1.00 |
|  | Agreement: κ  95% CI  Interpretation* | 0.21  0-0.46  very low | 0.38  0.11-0.66  low | 0.19  0.02-0.35  very low | 0.47  0.27-0.68  moderate | 0.43  0.26-0.61  moderate |

AUC: area under the curve; Sens: sensibility, Spe: specificity, NPV: negative predictive value, PPV: positive predictive value; κ; Cohen’s kappa coefficient. *Interpretation according to Landis and Koch, Biometrics (1977); 33: 159-174 (<https://doi.org/10.2307/2529310>). Bold values highlight significant differences between the values obtained using the supplier’s cut-off and those obtained using the optimized cut-off.
